# Living with congenital adrenal hyperplasia: insights on quality of life

**DOI:** 10.3389/fendo.2026.1795860

**Published:** 2026-03-11

**Authors:** Athanasia Bouliari, Louise Fleming, Ivora Hinton, Dina Matos, Jamilia McCoy, Lesley Holroyd, Karen Lin-Su

**Affiliations:** 1 New York-Presbyterian Hospital, Division of Pediatric Endocrinology, Weill Cornell Medicine, New York, NY, United States; 2 The University of Virginia, School of Nursing, Charlottesville, VA, United States; 3 Congenital Adrenal Hyperplasia Research Education and Support (CARES) Foundation, Union, NJ, United States

**Keywords:** adults, CAH, children, quality of life, survey

## Abstract

**Introduction:**

Understanding the quality of life (QoL) and factors associated with improved outcomes in individuals with classical congenital adrenal hyperplasia (CAH) can meaningfully inform clinical care.

**Methods:**

Adults and caregivers of children with classical CAH completed a cross-sectional mixed-methods survey distributed via the CARES (Congenital Adrenal Hyperplasia Research Education and Support) Foundation website. QoL was assessed using a CAH-specific QoL instrument adapted from the CAHQL. Domains included general health (GH), adrenal insufficiency (AI), glucocorticoid excess (GE), physical functioning (PF), mental health and cognition (MHC); adult surveys also included social functioning (SoF) and sexual functioning (SeF). Scores were transformed to a 0–100 scale, with higher scores indicating better QoL.

**Results:**

A total of 362 surveys were analyzed (response rate 15.7%), including 151 (41%) completed by adults, and 211 (59%) completed by caregivers of children. Among adult respondents, 71.8% were female, 76% resided in the United States (US), 58.1% had private insurance and 18.7% received care from CARES Comprehensive Care Center (CCC)-affiliated providers. Among children, 50% were female, 73% lived in the US, 66% had private insurance and 22.3% received CARES CCC-affiliated care. Adults demonstrated mid-range mean QoL scores across domains with a total mean score of 58.1 (SD 17.7). Children had significantly higher median scores across all five domains (p<0.001, p<0.001, p<0.001, p=0.009, p<0.001). In adults, older age was associated with worse PF and SeF (both p<0.01). Female sex was associated with worse GE and SeF scores (both p<0.01). Public insurance was associated with worse QoL across most domains except for GE and PF (p= 0.01, p= 0.05, p= 0.03, p= 0.02, p= 0.02). In children, older age was associated with worse QoL across domains except PF (p<0.001 for all domains). CARES CCC affiliation was associated with significantly better AI and PF scores (p=0.03, p=0.02) in adults. Finally, long-acting steroid use was associated with worse GE scores (p= 0.03).

**Conclusion:**

Adults with CAH experience poorer QoL compared to children. Older age, female sex and public insurance were associated with worse QoL, while CARES CCC affiliation and hydrocortisone use were associated with improved outcomes.

## Introduction

Congenital adrenal hyperplasia (CAH) is a rare, autosomal recessive condition caused by deficiencies in the enzymes involved in adrenal steroid hormone synthesis. The most common form, 21-hydroxylase (21OH) deficiency, results from pathogenic variants in the *CYP21A2* gene. The severity of the clinical presentation of CAH varies, depending on the extent of the 21-OH enzyme deficiency, leading to different degrees of mineralocorticoid and glucocorticoid deficiency as well as androgen excess. The severe form, known as classical CAH, is further categorized into salt-wasting (SW) and simple-virilizing (SV) types depending on whether mineralocorticoid deficiency is present. The milder form, non-classical (NC) CAH is characterized by sufficient mineralocorticoid and glucocorticoid synthesis, with androgen excess representing the primary hormonal imbalance (1).

Hormonal imbalances in classical CAH can lead to clinical manifestations such as skin hyperpigmentation, premature adrenarche, precocious puberty, advanced bone age with compromised final adult height, acne, and hirsutism. Individuals with CAH are at risk for adrenal crises, impaired fertility, cardiometabolic complications and decreased bone mineral density. In female (46, XX) classical patients, there is virilization to varying degrees of the external genitalia, and genital restorative surgery may be considered. Management of classical CAH requires frequent medical appointments and daily glucocorticoid replacement therapy with or without mineralocorticoid supplementation. All patients require stress dosing at times of illness, trauma, or surgery. Other adjunctive therapies such as crinecerfont, GnRH analogues, growth hormone, aromatase inhibitors and oral contraceptive pills may also be prescribed when indicated (2).

The medical and psychosocial challenges faced by patients with CAH and their caregivers, together with potential financial, sociocultural and health access barriers, can have a substantial impact on the overall quality of life (QoL) (3). Previous studies investigating the QoL in adult and pediatric patients with CAH, using different validated questionnaires, have reported that children and adolescents exhibit lower self-reported school domain QoL and a lower caregiver-reported psychosocial QoL, while findings among adults have been mixed (4–8). Poor disease control and medication adherence as well as clinical complications were associated with lower QoL scores in children and adolescents, while treatment with dexamethasone or prednisolone, increased adiposity and insulin resistance were associated with impaired QoL in adults with CAH (4, 5).

CARES Foundation is a non-profit organization that seeks to improve the lives of the CAH community through support, advocacy, education and research. CARES Foundation has currently designated nine centers of excellence called CAH Comprehensive Care Centers (CCCs) across the United States. They comprise multi-disciplinary teams of healthcare professionals who are experts or developing experts in the care of CAH with the goal of providing excellent care to patients with CAH from childhood to adulthood. Understanding the experiences and outcomes of individuals with classical CAH is essential to enhancing patient care and overall quality of life. This study aims to explore the demographic characteristics, self- and caregiver reported health data, and quality of life measures in both adult and pediatric patients with classical CAH and identify factors associated with improved quality of life.

## Methods

### Study design and participants

Data was collected from adults ≥ 18 years and caregivers of children < 18 years with classical CAH using a cross-sectional, mixed methods survey. Recruitment was conducted through CARES (Congenital Adrenal Hyperplasia Research Education and Support) Foundation, an international nonprofit patient support organization. An anonymous Qualtrics survey was distributed via CARES Foundation website (https://www.caresfoundation.org) and was available between September 18, 2025, to November 10, 2025. Patients and parents registered with CARES Foundation, representing approximately 11-12% of individuals with classical CAH in the United States (2,300 of an estimated 20,000 affected), as well as members of other international CAH support groups, received invitations and communications regarding participation in the study.

The study was approved by the Institutional Review Board (IRB) at University of Virginia and was conducted in accordance with the IRB guidelines and ethical standards. Informed consent was obtained from all participants prior to survey participation.

### Survey tool

An original survey was developed for this study and information on demographic characteristics (age, sex, gender, race, ethnicity, country, insurance status) and CAH-specific health data (CAH provider information, medication list) were collected. Provider information included whether participants were receiving care at a CARES CCC. Additionally, the survey incorporated a CAH-specific health-related QoL tool, adapted from the CAHQL, a validated instrument designed to assess disease specific health-related QoL outcomes in individuals with CAH aged 16 years and older, as previously described in the literature (9).

Domains within the QoL tool included general health, adrenal insufficiency, glucocorticoid excess, physical functioning, mental health and cognition for both adult and caregiver surveys. Items within each domain were identical to those in the CAHQL instrument and consistent across both survey versions to ensure comparability, with only minor wording modifications in the caregiver survey to reflect caregivers’ perspectives on their child’s symptoms and experiences. The adult survey additionally included social and sexual functioning domains, which were omitted from the pediatric surveys due to lack of relevance (see **Supplementary Material**). Response options for each item were simplified to a uniform 5-point frequency scale (Never, Sometimes, About half the time, Most of the time, Always) to improve clarity and reduce respondent burden. This differed from the original CAHQL, which uses variable 5–6 point response formats across items (9). Internal consistency of the adapted instrument was not formally assessed.

Participants were also asked to provide free-text responses to the question *“What would you like your healthcare provider to know about what it is like living with CAH?”* or, for caregivers, *“What do you want your child’s doctor to know about what it’s like for your child to live with CAH?”.* This allowed respondents to share, in their own words, aspects of their lived experience that they felt were insufficiently captured by structured survey items or routine clinical encounters. (See **Supplementary Material**).

### Analysis

The CAH-specific health-related quality-of-life tool consisted of 5-point Likert-scale response options. The raw score of each item was transformed to a 0 to 100 scale, and reverse coded when needed so higher domain scores represented a better quality of life. The domain scores were calculated by adding the scores of the individual items and dividing by the total number of domain items.

SPSS Version 31 was used to perform the statistical analysis. Descriptive statistics were calculated for age, sex, country of residence, insurance status, and CARES CCC affiliation as well as the domain scores. Kolmogorov-Smirnov tests were used to evaluate the normality of the distribution of the domain scores. Domain scores are reported as medians with interquartile ranges for non-normally distributed variables and means with standard deviations for normally distributed variables. Comparisons between groups were performed using independent samples t-tests or Mann-Whitney U tests, with p-values reflecting differences in mean or median domain scores. Associations between age and quality-of-life domain scores were assessed using Spearman correlation coefficients. Correlation coefficients (ρ) and corresponding p-values are reported.

Responses to a single open-ended survey question were reviewed using a descriptive, exploratory approach. Because responses were collected through a cross-sectional survey and not through in-depth interviews or iterative qualitative methods, the analysis was conducted to summarize and organize recurring concepts rather than to develop theory or achieve thematic saturation.

## Results

A total of 362 surveys were collected, representing an overall response rate to 15.7%. Of these, 151 (41%) were completed by adults with CAH and 211 (59%) were completed by caregivers of children with CAH. Among adult participants, 42 (28.2%) were male and 107 (71.8%) were female, with a mean age of 39.7 years (SD 15.1). Most adults were United States (US) residents (N = 114, 76%) while 36 (24%) lived outside the US. Private insurance was reported by 86 (58.1%), whereas 62 (41.9%) had public insurance. Only 28 (18.7%) of the adult participants had a health care provider affiliated with a CARES CCC. (**Table 1**) Among children, 105 (50%) were male. The mean age was 95.4 months (SD 63.2) and 154 (73%) resided in the US. Most children (N = 138, 66%) had private insurance and 47 (22.3%) received care at a CARES CCC. (**Table 2**) In terms of steroid treatment, 80 (61.5%) adults were on hydrocortisone, with 50 (38.5%) being on long-acting steroid (prednisone/dexamethasone) therapy. Only 5 (3%) children were receiving treatment with long-acting steroids.

**Table 1:** Demographic information (adults).

Demographic Characteristic	N (%)	Mean	Min-Max
What is your sex?			
Male XY	42 (28.2%)		
Female XX	107 (71.8%)		
Total	149		
What is your age in years?			
	141		
		39.7 (15.1)	18 - 77
Where do you live (United States or Outside of US)?			
United States	114 (76%)		
Outside of US	36 (24%)		
Total	150		
Residence Country			
US	114 (76%)		
Canada	5 (3.3%)		
France	9 (6%)		
Germany	9 (6%)		
Other Europe Country	11 (7.3%)		
Other Country	2 (1.3%)		
Total	150		
Insurance Status			
Private	86 (58.1%)		
Public	62 (41.9%)		
Total	148		
Is the health care provider affiliated with a CARES Comprehensive Care Center?			
Yes	28 (18.7%)		
No	122 (81.3%)		
Total	150		

**Table 2:** Demographic information (children).

Demographic Characteristic	N (%)	Mean	Min-Max
What is the sex of your child living with CAH?			
Male XY	105 (50%)		
Female XX	105 (50%)		
Total	210		
What is the age of your child in months?			
	207		
		95.4 (63.2)	1 - 215
Where do you live (United States or Outside of US)?			
United States	154 (73%)		
Outside of US	57 (27%)		
Total	211		
Residence Country Categories			
US	154 (73%)		
Canada	11 (5.2%)		
France	14 (6.6%)		
Germany	13 (6.2%)		
Other Europe	11 (5.2%)		
Other Country	8 (3.8%)		
Total	211		
Insurance Status			
Private	138 (66%)		
Public	71 (34%)		
Total	209		
Is the health care provider affiliated with a CARES Comprehensive Care Center?			
Yes	47 (22.3%)		
No	164 (77.7%)		
Total	211		

Adult respondents had a mean total QoL score of 58.1 (SD: 17.7) with domain-specific scores shown in **Table 3**. Children had a higher mean total QoL score of 73.5 (SD 13.6) with domain scores presented in **Table 4**. Overall, adults demonstrated significantly more impaired QoL across all domains when compared to children. Median domain scores for children versus adults were as follows: general health 80 (IQR: 60, 95) vs 65 (IQR: 38.8, 80), p<0.001, adrenal insufficiency 72.2 (IQR: 63.2, 80.6) vs 52.8 (IQR: 40.3, 66.7), p<0.001, glucocorticoid excess 91.7 (IQR: 83.3, 100) vs 62.5 (IQR: 41.7, 79.2), p<0.001, physical functioning 66.7 (IQR: 58.3, 79.2) vs 58.3 (IQR: 37.5, 78.1), p= 0.009 and mental health and cognition 80.6 (IQR: 63.9, 91.7) vs 61.1 (IQR: 41.7, 70.8), p<0.001. (**Table 5**).

**Table 3:** Quality-of-life domain scores for adults.

Domain	N	M (SD)	Median (IQR)	Minimum-maximum
Total	131	58.1 (17.7)		45.9 - 60.6
General health	146	58.2 (25.6)	65.0 (38.8, 80.0)	0 – 100
Adrenal insufficiency	149	53.5 (19.2)	52.8 (40.3, 66.7)	2.8 – 94.4
Glucocorticoid excess	148	60.2 (22.4)	62.5 (41.7, 79.2)	12.5 – 100
Physical functioning	148	57.5 (25.7)	58.3 (37.5, 78.1)	0 – 100
Mental health and cognition	145	56.6 (19.6)	61.1 (41.7, 70.8)	0 – 94.4
Social functioning	144	67.6 (25.5)	75.0 (55.0, 90.0)	0 – 100
Sexual functioning	141	56.1 (31.9)	62.5 (25.0, 81.3)	0 – 100

**Table 4:** Quality-of-life domain scores for children.

Domain	N	M (SD)	Median (IQR)	Minimum-maximum
Total	180	73.5 (13.6)		29.3 - 93.3
General health	204	74.7 (21.6)	80.0 (60, 95)	5 – 100
Adrenal insufficiency	202	69.5 (14.3)	72.2 (63.2, 80.6)	25 – 97.2
Glucocorticoid excess	201	87.7 (15.8)	91.7 (83.3, 100)	25 – 100
Physical functioning	191	64.5 (18.2)	66.7 (58.3, 79.2)	0 – 91.7
Mental health and cognition	192	76.1 (19.2)	80.6 (63.9, 91.7)	0 – 100

**Table 5:** Comparison of quality-of-life domain scores between adults and children.

Domain	Data	N	Median (IQR)	df	z	p
General health	Adult	146	65.0 (38.8, 80.0)	348	6.07	<.001*
General health	Children	204	80.0 (60.0, 95.0)			
Adrenal insufficiency	Adult	149	52.8 (40.3, 66.7)	349	7.87	<.001*
Adrenal insufficiency	Children	202	72.2 (63.2, 80.6)			
Glucocorticoid excess	Adult	148	62.5 (41.7, 79.2)	347	11.15	<.001*
Glucocorticoid excess	Children	201	91.7 (83.3, 100)			
Physical functioning	Adult	148	58.3 (37.5, 78.1)	337	2.61	0.009*
Physical functioning	Children	191	66.7 (58.3, 79.2)			
Mental health and cognition	Adult	145	61.1 (41.7, 70.8)	335	8.61	<.001*
Mental health and cognition	Children	192	80.6 (63.9, 91.7)			

The asterisk indicates statistical significance.

In the adult cohort, there was a significant negative correlation with age in the physical and sexual functioning domains (Spearman’s ρ = -0.27, *p* < 0.001 and ρ = −0.29, *p* < 0.001), whereas in the pediatric cohort, there was a significant negative correlation with age across all domains except for physical functioning (general health ρ=-0.29, p<0.001, adrenal insufficiency ρ=-0.41, p<0.001, glucocorticoid excess ρ=-0.38, p<0.001, mental health and cognition ρ=-0.33, p<0.001). Among adults, female sex was also associated with significantly lower scores in the glucocorticoid excess domain [58 (IQR: 42, 71) vs 75 (IQR: 54, 88), p<0.01] and sexual functioning domain [56 (IQR: 19, 75) vs 81 (IQR: 52, 100), p<0.01].

Public insurance was another factor found to be associated with poorer QoL in adults. Specifically, adults with public insurance had significantly lower scores in the general health, adrenal insufficiency, mental health and cognition, social and sexual functioning domains. (**Table 6**) When the analysis was limited to adults residing in the US, those with public insurance additionally demonstrated worse physical functioning compared with those with private insurance (**Table 7**). Public insurance status was not associated with worse QoL scores in the overall pediatric cohort. However, pediatric patients residing in the US with public insurance had significantly lower general health scores [62.5 (IQR: 48.8, 76.3) vs 80 (IQR: 60, 95), p=0.01].

**Table 6:** Comparison of quality-of-life domain scores between adults with public and private insurance in the complete adult cohort.

Domain	Insurance status	N	M (SD) or median (IQR)	df	t or z	p
T-test						
Glucocorticoid excess	Private	85	61.18 (21.0)	144	0.85	0.4
Glucocorticoid excess	Public	61	57.99 (24.1)			
Mann U						
General health	Private	82	70 (54, 80)	141	-2.79	0.01 *
General health	Public	61	45 (30, 75)			
Adrenal insufficiency	Private	85	58 (44, 69)	144	-1.96	0.05 *
Adrenal insufficiency	Public	61	50 (39, 64)			
Physical functioning	Private	84	65 (42, 79)	143	-1.89	0.06
Physical functioning	Public	61	54 (33, 75)			
Mental health and cognition	Private	83	64 (53, 72)	140	-2.16	0.03 *
Mental health and cognition	Public	59	53 (36, 69)			
Social functioning	Private	82	80 (59, 90)	139	-2.30	0.02 *
Social functioning	Public	59	65 (45, 80)			
Sexual functioning	Private	81	69 (41, 84)	137	-2.33	0.02 *
Sexual functioning	Public	58	50 (19, 77)			

The asterisk indicates statistical significance.

**Table 7:** Comparison of quality-of-life domain scores between adults residing in the Unite States with public versus private insurance.

Domain	Insurance status	N	M (SD) or median (IQR)	df	t or z	p
T-test						
Glucocorticoid excess	Private	75	60.83 (21.07)	108	1.11	0.27
Glucocorticoid excess	Public	35	55.83 (24.05)			
Physical functioning	Private	74	60.59 (23.35)	107	2.83	0.01*
Physical functioning	Public	35	46.43 (26.51)			
Mann U						
General health	Private	73	70 (48, 80)	106	-3.79	< 0.001 **
General health	Public	35	40 (25, 55)			
Adrenal insufficiency	Private	75	58 (44, 69)	108	-3.09	< 0.01 *
Adrenal insufficiency	Public	35	44 (33, 58)			
Mental health and cognition	Private	73	64 (53, 71)	105	-2.96	< 0.01 *
Mental health and cognition	Public	34	49 (33, 64)			
Social functioning	Private	73	80 (60, 90)	105	-2.62	0.01 *
Social functioning	Public	34	63 (40, 81)			
Sexual functioning	Private	71	69 (44, 88)	103	-2.82	0.01 *
Sexual functioning	Public	34	41 (13, 77)			

The asterisk indicates statistical significance.

In the pediatric cohort, residence outside the US was associated with improved general health outcomes [85 (IQR: 65, 95) vs 75 (IQR: 55, 90), p=0.04]. Adults residing outside the US demonstrated better general health, physical functioning, mental health and cognition and adrenal insufficiency scores, though the differences were not statistically significant. (**Table 8**).

**Table 8:** Comparison of quality-of-life domain scores between adults residing in the United States and outside the united states.

Domain	Where do you live?	N	M (SD) or median (IQR)	df	t or z	p
T-test						
Adrenal insufficiency	United States	113	51.84 (38.9)	146	-1.84	0.67
Adrenal insufficiency	Outside of US	35	58.65 (44.4)			
Mann U						
General health	United States	111	60.0 (35.0, 80.0)	143	1.31	0.19
General health	Outside of US	34	70.0 (47.5, 81.3)			
Glucocorticoid excess	United States	112	62.5 (41.7, 78.1)	145	0.60	0.55
Glucocorticoid excess	Outside of US	35	62.5 (41.7, 79.2)			
Physical functioning	United States	112	58.3 (37.5, 75.0)	145	1.28	0.20
Physical functioning	Outside of US	35	62.5 (54.2, 83.3)			
Mental health and cognition	United States	110	58.3 (43.8, 66.7)	142	1.10	0.27
Mental health and cognition	Outside of US	34	65.3 (40.3, 75.7)			
Social functioning	United States	110	75.0 (55.0, 90.0)	141	-0.42	0.68
Social functioning	Outside of US	33	75.0 (47.5, 90.0)			
Sexual functioning	United States	107	62.5 (25.0, 81.3)	138	-0.11	0.91
Sexual functioning	Outside of US	33	56.3 (25.0, 81.3)			

Adults receiving care from a CARES CCC-affiliated provider had significantly higher adrenal insufficiency [60.6 (SD: 47.2) vs 51.8 (SD: 19.8), p=0.03] and physical functioning [67.9 (SD: 20.5) vs 55.1 (SD: 26.4), p=0.02] scores, indicating fewer adrenal insufficiency–related symptoms and better physical functioning. Children with a CARES CCC-affiliated provider had significantly better general health scores [90 (IQR: 21.1, 90) vs 75 (IQR: 21.6, 75), p=0.03]. (**Tables 9**, **10**). These analyses included all adult and pediatric participants, including both US-based and international respondents. In the analyses examining the impact of the type of steroid regimen on adult QoL, those treated with long-acting steroids had significantly lower glucocorticoid excess scores [55.6 (SD 22.8) vs 64.3 (SD.21.4), p=0.03] compared with hydrocortisone. (**Table 11**).

**Table 9:** Comparison of quality-of-life domain scores between adults with and without a CARES comprehensive care center affiliated provider.

Domain	Is the health care provider affiliated with a CARES comprehensive care center?	N	M (SD) or median (IRQ)	df	t or z	p
T-test						
Adrenal insufficiency	Yes	27	60.6 (47.2)	146	2.18	0.03 *
Adrenal insufficiency	No	121	51.8 (19.8)			
Physical functioning	Yes	27	67.9 (20.5)	145	2.37	0.02 *
Physical functioning	No	120	55.1 (26.4)			
Mann U						
General health	Yes	27	70 (40, 80)	143	-1.22	0.22
General health	No	118	60 (35, 76)			
Glucocorticoid excess	Yes	26	69 (46, 79)	145	-0.83	0.41
Glucocorticoid excess	No	121	58 (42, 79)			
Mental health and cognition	Yes	25	64 (57, 71)	142	-1.81	0.07
Mental health and cognition	No	119	58 (39, 72)			
Social functioning	Yes	26	73 (60, 86)	141	-0.28	0.78
Social functioning	No	117	75 (50, 90)			
Sexual functioning	Yes	25	50 (22, 78)	138	0.73	0.47
Sexual functioning	No	115	63 (31, 81)			

The asterisk indicates statistical significance.

**Table 10:** Comparison of quality-of-life domain scores between children with and without a CARES comprehensive care center affiliated provider.

Domain	Is the health care provider affiliated with a CARES comprehensive care center?	N	median (IQR)	df	z	p
General health	Yes	45	90.0 (21.1, 90.0)	202	-2.14	0.03 *
General health	No	159	75.0 (21.6, 75.0)			
Adrenal insufficiency	Yes	44	72.2 (13.2, 72.2)	200	-0.16	0.87
Adrenal insufficiency	No	158	72.2 (14.6, 72.2)			
Glucocorticoid excess	Yes	44	95.8 (17.5, 95.8)	199	-1.29	0.20
Glucocorticoid excess	No	157	91.7 (15.3, 91.7)			
Physical functioning	Yes	41	66.7 (16.2, 66.7)	189	-0.42	0.68
Physical functioning	No	150	66.7 (18.8, 66.7)			
Mental health and cognition	Yes	43	83.3 (19.5, 83.3)	190	-0.86	0.39
Mental health and cognition	No	149	77.8 (19.1, 77.8)			

The asterisk indicates statistical significance.

**Table 11:** Comparison of quality-of-life domain scores between adults on hydrocortisone versus long-acting glucocorticoids (dexamethasone and prednisone).

Domain	Medications	N	M (SD) or median (IQR)	df	t	p
T-test						
Glucocorticoid excess	Hydrocortisone	78	64.3 (21.4)	126	2.18	0.03*
Glucocorticoid excess	Prednisone/dexamethasone	50	55.6 (22.8)			
Physical functioning	Hydrocortisone	78	53.6 (24.5)	125	-1.50	0.14
Physical functioning	Prednisone/dexamethasone	49	60.5 (27.0)			
Mann Whitney U test						
General health	Hydrocortisone		65.0 (37.5 - 80.0)	123	0.18	0.86
General health	Prednisone/dexamethasone		62.5 (31.3 - 80.0)			
Adrenal insufficiency	Hydrocortisone		55.6 (38.9 - 63.9)	126	0.60	0.55
Adrenal insufficiency	Prednisone/dexamethasone		52.8 (43.1 - 70.8)			
Mental health and cognition	Hydrocortisone		58.3 (41.7 - 67.4)	123	0.95	0.34
Mental health and cognition	Prednisone/dexamethasone		63.9 (41.7 - 72.2)			
Social functioning	Hydrocortisone		70.0 (55.0 - 85.0)	122	0.40	0.69
Social functioning	Prednisone/dexamethasone		80.0 (52.5 - 90.0)			
Sexual functioning	Hydrocortisone		62.5 (31.3 - 81.3)	119	0.18	0.86
Sexual functioning	Prednisone/dexamethasone		62.5 (31.3 - 81.3)			

The asterisk indicates statistical significance.

Among adult participants, 121 provided a substantive response to the question *“What would you like your healthcare provider to know about what it is like living with CAH?”*, while among caregivers 102 responded to the question *“What do you want your child’s doctor to know about what it’s like for your child to live with CAH?”*. Responses from both groups were organized in four main domains that included core lived experiences, psychological and emotional impact, treatment and daily management and healthcare system experiences. These themes are further detailed in **Table 12**.

**Table 12:** Comparison of adult and caregiver-reported domains in congenital adrenal hyperplasia.

Domain	Adults with CAH (n=121)	Caregivers of children with CAH (n=102)
Core lived experience	**Pervasive physical burden and unpredictability**: Chronic fatigue, low energy, and unpredictable symptoms despite “normal” laboratory values; physiological vigilance to avoid decompensation or adrenal crisis.	**Constant vigilance**: Continuous monitoring for illness or stress, especially in children unable to articulate symptoms; persistent anticipatory anxiety, particularly overnight, during illness, school, or travel.
Psychological/emotional impact	**Psychological and emotional impact**: Anxiety, depression, emotional lability, shame related to genital appearance (females), sexual functioning, infertility, or weight; long-term effects on self-esteem, relationships, and mental health often perceived as underrecognized by providers.	**Behavioral and developmental impact**: Anxiety, irritability, sleep disturbances, emotional dysregulation; difficulty distinguishing typical development from CAH-related behaviors; concerns about stigma, body image, growth, and feeling different from peers.
Treatment and Daily management	**Challenges of disease management and treatment burden**: Strict medication schedules, stress-dosing decisions, side effects of long-term glucocorticoid use, uncertainty around optimal dosing, and concern for long-term sequelae (e.g., osteoporosis, metabolic effects).	**Complexity of medical management across daily life settings**: Logistical challenges of managing medication, stress dosing, and emergency preparedness across schools, childcare, sports, and travel; reliance on others to administer life-saving treatment; desire for improved delivery systems.
Healthcare system experience	**Gaps in healthcare understanding, personalization, and coordination**: Feeling dismissed when symptoms do not align with laboratory values; fragmented care; lack of provider understanding outside specialty endocrinology; desire for individualized, patient-centered care.	**Need for provider partnership and holistic care**: Frustration with minimization of concerns related to fatigue, growth, weight, or emotional stress; value placed on providers who listen, individualize care, and coordinate across systems; concerns about limited CAH knowledge among non-endocrine and emergency providers.

Bold text is used to further categorize responses.

## Discussion

To our knowledge, this is the first study to assess quality of life (QoL) in both adult and pediatric patients with classical CAH using a CAH-specific health-related QoL instrument adapted from the validated CAHQL. Individuals with CAH may experience disease and treatment related adverse health outcomes, such as symptoms of adrenal insufficiency, glucocorticoid excess, and impaired sexual functioning, that uniquely and substantially impact their QoL. In contrast to commonly used generic health related QoL measures for adult and pediatric patients (e.g. SF-36, PedsQL) which include domains such as physical, social, and emotional functioning and mental health, the questionnaire used in this study specifically captured additional CAH-specific domains, such as adrenal insufficiency, glucocorticoid excess, and sexual functioning.

Overall, adult participants demonstrated relatively poor quality of life, with scores in the mid-range across domains. Lower scores were observed in the adrenal insufficiency, mental health and cognition and sexual functioning domains, while higher scores were noted in social functioning. This is consistent with prior literature indicating that adults with CAH experience significantly impaired quality of life compared with matched controls across multiple domains, including general health, physical and social functioning, and emotional and mental health (10–12).

Overall pediatric data showed higher mean scores across all evaluated domains compared to adults. Lower overall scores were observed in the physical functioning and adrenal insufficiency domains with higher scores in the glucocorticoid excess domain. This is in contrast with prior studies reporting higher parent-reported physical domain mean scores and no significant differences in parent-reported physical quality of life (4) compared with matched controls, though direct comparisons are limited due to the use of different assessment instruments. This may reflect the inclusion of more CAH-specific physical functioning questions in the CAHQL compared with the PedsQL, including items related to activity in heat, hydration during activity, and recovery time.

When compared to the adult results, children appear to have significantly higher QoL scores across all five domains. This finding may be explained by treatment differences between children and adults, as children with CAH are more commonly managed with hydrocortisone rather than long-acting glucocorticoids, which carry a greater risk of adverse effects related to glucocorticoid excess. Supporting this, our study observed a low rate of long-acting steroid use in the pediatric population, and adults treated with long-acting glucocorticoids demonstrated worse glucocorticoid excess outcomes compared to those receiving hydrocortisone. Children are also less likely to experience CAH- or treatment-related adverse outcomes, such as cardiometabolic complications, osteopenia, osteoporosis and compromised fertility, which most commonly emerge later in life and may negatively impact QoL. Pediatric results should be interpreted cautiously because data were reported by caregivers rather than directly by patients, which may introduce limitations in the assessment of domains such as mental health and cognition.

In the adult cohort, females reported significantly greater concerns related to glucocorticoid excess compared with males. This is consistent with prior studies showing that corticosteroid use is associated with higher odds of metabolic syndrome in women, but not men (13) and may reflect by sexually dimorphic effects of glucocorticoids previously described in the literature (14). This is further supported by the lack of this difference in the pediatric data, suggesting that hormonal changes occurring after puberty may modulate susceptibility to glucocorticoid-related adverse effects. Females also reported significantly worse sexual functioning when compared to males, aligning with prior studies showing that women more frequently report being unhappy about their sex life when compared to men (10). This may reflect the combined impact of anatomic changes and hormonal factors affecting women with CAH.

Public insurance was associated with poorer QoL outcomes across most domains in the adult cohort, an association not observed in the pediatric cohort. When the analysis was restricted to individuals residing in the US, differences between privately and publicly insured were more pronounced and encompassed all domains except for glucocorticoid excess. This disparity may reflect challenges commonly faced by adults with public insurance in the US, including inconsistent, income-based or inadequate coverage, higher out-of-pocket costs and reduced access to care (particularly mental health services). In contrast, children with public insurance in the US may experience more comprehensive and stable coverage through established programs, such as Medicaid and CHIP (Children’s Health Insurance Program), which could mitigate impacts on quality of life. This interpretation is further supported by the higher overall scores observed in most domains among adults residing outside of the US, although these differences were not statistically significant.

Another major finding of our study was that affiliation with a CARES Comprehensive Care Center (CARES CCC) was associated with significantly better physical functioning and fewer concerns related to adrenal insufficiency in the adult cohort as well as improved general health scores in the pediatric cohort. Overall, mean QoL scores were higher across most domains among patients affiliated with a CARES CCC in both children and adults, with the exception of social and sexual functioning in adults. The lack of statistical significance in other domains may be attributable to the smaller sample size within the CARES CCC affiliation groups. Improved QoL outcomes in this group are likely related to access to comprehensive, multi-disciplinary care, including adult and pediatric endocrinologists, urologists, reproductive health specialists, geneticists, nutritionists, and mental health providers with expertise in the management of CAH.

The exploratory synthesis of adult self-reported and caregiver-reported experiences from the open-ended survey question highlights both shared challenges and developmentally distinct aspects of living with CAH. Across both groups, respondents emphasized that QoL extends beyond biochemical disease control to encompass physical symptoms, emotional stress, complex management demands, and gaps in healthcare understanding. These findings suggest that, while responsibility for condition management shifts from caregiver-led vigilance to self-management across the lifespan, the demands of disease management are not eliminated but transformed. Adults carry the internalized consequences of years of disease management, while caregivers shoulder considerable external responsibilities. These findings underscore the need for a holistic, lifespan-oriented approach to CAH care that integrates physical, psychological, and social dimensions. Given that these data derive from a single open-ended survey question rather than a dedicated qualitative study, future in-depth qualitative research is warranted to further elucidate these experiences and inform targeted interventions to improve QoL across the lifespan.

One limitation of this study, is that participants were recruited exclusively through CARES Foundation, which represents a small proportion (11-12%) of individuals with classical CAH in the US (approximately 2,300 registered with CARES out of an estimated 20,000 affected in U.S., based on U.S. population of 340 million and prevalence of 1 in 16,000 to 1 in 17,000) (15),, though the number of living classical CAH patients is likely overestimated since treatment was not available before the 1950s, so patients older than 75 would not have survived. In addition, only 15.7% of those invited completed the survey, potentially introducing selection bias. More specifically, it is unclear whether survey respondents were more motivated, with greater access to information and higher health literacy than the broader CAH population, or conversely, whether individuals experiencing more CAH related complications were more likely to participate in a QoL survey. In addition, although the QoL instrument was adapted from a previously validated measure, its internal consistency was not formally evaluated in this sample. Finally, while no matched healthy control group was included and standardized reference scores are lacking for the CAH-specific QoL instrument, these limitations are common in studies of rare diseases and do not preclude interpretation of the observed results.

Strengths of the study include the representation of both adults and children, males and females across a broad age range, a well-balanced distribution of privately and publicly insured participants, and inclusion of data from both the U.S. and international cohorts. In addition, the inclusion of the open-ended survey questions, allowed respondents to provide detailed, personal insights and describe their experiences in their own words.

In conclusion, this is the first study to assess the QoL of individuals, both adults and children, with CAH using a disease specific questionnaire, revealing an overall impaired QoL, particularly among adults. Modifiable factors potentially associated with better QoL included affiliation with a CARES CCC, which appeared to be beneficial for physical functioning and adrenal insufficiency in adults, and the use of hydrocortisone which can decrease concerns related to glucocorticoid excess associated with long-acting steroids. Non modifiable factors potentially associated with poorer QoL included older age, female sex, and public insurance status. These findings highlight the need for provider awareness and increased attentiveness to QoL challenges in higher-risk groups, including older individuals, females, and those with public insurance, with consideration of modifiable contributors through referrals to CARES Comprehensive Care Centers and optimization of steroid therapy. However, interpretation should be cautious, as respondents were recruited through CARES Foundation, and it is unclear how representative they are of the broader CAH population in the US or globally.

## Data Availability

The raw data supporting the conclusions of this article will be made available by the authors, without undue reservation.
