# Supplementary material for: Living with congenital adrenal hyperplasia: insights on quality of life
Source: Front Endocrinol (Lausanne). 2026 Mar 11;17:1795860. doi: 10.3389/fendo.2026.1795860 (PMC13012940; doi:10.3389/fendo.2026.1795860)
Supplement: Supplementary file 1 [file DataSheet1.pdf]

CAH Survey Updated

Start of Block: Introduction

Thank you for your willingness to participate in this study. This survey is intended for caregivers/parents/guardians of children between the ages of birth-18 years of age and adults with classic (salt-wasting) congenital adrenal hyperplasia (CAH). If you don't feel like you qualify to participate in this study, please contact me before continuing the survey. This survey should take approximately 20-30 minutes to complete. Your insights are valuable in helping us better understand the diverse perspectives and experiences within the Congenital Adrenal Hyperplasia (CAH) community. This survey is designed to explore health-related and quality of life information; differences between U.S. and international participants; and patient experiences with adrenal crisis events and CAH treatment/ management. Please answer the questions to the best of your knowledge and remember that the answers to this survey are confidential. **Please understand that by completing this survey, you are consenting to participate in this study.** Thank you. Louise Fleming, PhD, MSN-Ed, RN, FAAN lfleming@virginia.edu Let's get started!

By continuing, I consent to participate in this survey. Do you consent to participate?

- o Yes, I consent — Continue to the survey. (1)
- o No, I do not consent — You will now exit the survey. Thank you for your time. (2)

Skip To: End of Survey If By continuing, I consent to participate in this survey. Do you consent to participate? = No, I do not consent — You will now exit the survey. Thank you for your time.

Skip To: QID62 If By continuing, I consent to participate in this survey. Do you consent to participate? = Yes, I consent — Continue to the survey.

Which statement best describes your relationship with classical/salt wasting CAH?

- o I am 18 or older living with CAH (1)
- o I am a caregiver of a child younger than 18, diagnosed with classical/salt wasting CAH (2)
- o Neither of the options above apply to me. (3)

Start of Block: Caregiver_Questions

- Caregiver or parent age? ------------
- Caregiver or parent gender?
  - o Male (1)
  - o Female (2)
  - o Non-binary / third gender (3)
  - o Prefer not to say (4)

Start of Block: General_Demographic

- **Please enter the date of birth of the person diagnosed with CAH (Congenital Adrenal Hyperplasia) in the following format**: MM/DD/YYYY (e.g., 01/01/1991).
- **Race of person with CAH? Select all that apply.**

- American Indian (1)
- Asian (2)
- Native Hawaiian (3)
- Black/African American (4)
- White American (5)
- other-please specify (6) ____
- I don't want to answer (7)

- **Ethnicity of person with CAH (Hispanic/Latino or Not Hispanic/Latino):**
  - o Hispanic/Latino (1)
  - o Not Hispanic/Latino (2)
  - o I do not want to answer (3)

Start of Block: Gender_Sex Parent response

- **What is the sex of your child living with CAH?**
  - o Male XY (1)
  - o Female XX (2)
  - o Prefer not to say (4)

Start of Block: Provider and resources parent response

- **Does your child see a pediatric endocrinologist to manage and treat CAH?**
  - o Yes (1)
  - o No (2)

Display this question: If Does your child see a pediatric endocrinologist to manage and treat CAH? = Yes

- **If yes, do you consider your child's provider to be an expert in CAH management?**

o Yes (1)

o No (2)

Display If Does your child see a pediatric endocrinologist to manage and treat CAH? = Yes

- **Approximately how many miles do you travel to see your child's endocrinologist? ---**

Display this question: If Does your child see a pediatric endocrinologist to manage and treat CAH? = No

- **If no, what type of healthcare provider treats your child's CAH?**
  - o General pediatrician (1)
  - o Family practice physician (2)

- Nurse practitioner (3)
- Other (4) ____

- **Is the health care provider who manages and treats your child CAH affiliated with a CARES Comprehensive Care Center?**
  - Yes (1)
  - No (2)

Display this question: If Is the health care provider who manages and treats your child CAH affiliated with a CARES Compreh... = Yes

- **If you selected yes, please chose the facility or facilities they are affiliated with.**
  - New York-Presbyterian/Weill Cornell Medical Center (1)
  - Children's Hospital Los Angeles / Keck School of Medicine (2)
  - Riley Hospital for Children/ Indiana University Health – Indianapolis IN (3)
  - Rutgers-Robert Wood Johnson Medical School (RWJMS), Child Health Center of New Jersey (CHINJ) (4)
  - Children's Health Medical Center/UT Southwestern Medical Center – Dallas, Texas (5)
  - Children's Hospital of Philadelphia/Penn Med – Philadelphia, PA (6)
  - Seattle Children's Hospital and University of Washington Medical Center – Seattle, Washington (7)
  - Cook Children's Health Care System – Fort Worth, Texas (8)
  - Other (9) ____

- **How far from your home is the hospital where you would take your child if he/she were in adrenal crisis?**
  - Less than 5 miles (Less than 8 km) (1)
  - 5–10 miles (8–16 km) (2)
  - 10–20 miles (16–32 km) (4)
  - 20–50 miles (32–80 km) (5)
  - More than 50 miles (More than 80 km) Please specify (6) --------------

Start of Block: Geography_and_finance_parent_response

- **Where do you live (city/state/country or province/territory/city/country)?**
  - United States US, please specify (1)
  - Outside of United states US, please specify (2)

Display this question: If Where do you live (city/state/country or province/territory/city/country)? = United States US, please specify

- **Approximate gross yearly family income (before taxes):**
    - Less than $20,000 (1)
    - $20,000 to $29,999 (2)
    - $30,000 to $39,999 (3)
    - $40,000 to $49,999 (4)
    - $50,000 to $59,999 (5)
    - $60,000 to $69,999 (6)
    - $70,000 to $79,999 (7)
    - $80,000 to $89,999 (8)
    - $90,000 to $99,999 (9)
    - $100,000 or more (10)
    - I'm not sure (11)
- **How do you pay for your child's medical care, choose all that apply?**
    - Private insurance through your job (1)
    - National Health Care (ex. UK and much of Europe) (2)
    - Medicare/Medicaid (US) (3)
    - Marketplace/Affordable Care Act (US) (4)
    - Other (5) ____

Start of Block: Medical Information_ parent response

- **To the best of your knowledge, how many times per year does your child have an adrenal crisis? ------------**
- **Has your child had a Bone density or DEXA scan?**
    - Yes (1)
    - No (2)

Display this question: If Has your child had a Bone density or DEXA scan? = Yes

- **Has your child been told that they have problems with their bones (ex. osteoporosis/osteopenia/bone fractures)?**
    - Yes, please specify (2) ____
    - No (4)
- **Does your child have any other medical conditions, including but not limited to hypertension, high blood pressure, or diabetes? If yes, please list them below.**

- Yes, please specify (4) ____
- No (5)

- **Please list all the medications your child is currently taking related to their CAH (eg fludrocortisone/Florinef, hydrocortisone/Cortef/Alkindi, prednisone, crinecerfont/Crenessity). --**

Start of Block: Solu-Cortef_parent response

- **Does your child have a prescription for injectable hydrocortisone (also known as Solu-Cortef)?**
    - Yes (1)
    - No (2)

Skip To: End of Block If Does your child have a prescription for injectable hydrocortisone (also known as Solu-Cortef)? = No

- **Which statement best fits how you obtained your child's prescription for injectable hydrocortisone?**
    - My child' s doctor prescribed it at diagnosis without me saying anything (1)
    - Sometime after diagnosis I had to ask my child's physician for the prescription of injectable hydrocortisone (2)
    - My child's physician prescribed injectable hydrocortisone for my child eventually without my asking for it (3)
    - Your child had to change physicians in order to receive the prescription for injectable hydrocortisone (4)
    - other (5) ____

- **Has your child's physician OR one of their staff (such as a nurse) ever demonstrated to you how to correctly administer a shot of injectable hydrocortisone to your child?**
    - Yes (1)
    - No (2)
    - I cant remember (3)

- **Have you filled your child's Solu-Cortef prescription? If not, what was the reason? (ex. I didn't want to use it, I didn't have transportation, I didn't know how to use it)?**
    - Yes (1)
    - No, please specify (2) ____

- **Does your child consistently have a vial of injectable hydrocortisone readily available for an emergency?(for example, for school, in the home, at daycare, in the car, etc.)**
    - No (1)

- Yes (2)

- **Does your child ever stress dose for psychological/emotional situations such as feeling depressed or experiencing a life changing event (death, illness in a family member) or taking exams, if yes please list example situations?**
    - Yes, please specify (2) ____
    - No (3)

Display this question: If Does your child ever stress dose for psychological/emotional situations such as feeling depressed... = Yes, please specify

- **If so, how many times per month would you say your child does this? If more than four times, please describe.**
    - Once a month (1)
    - Twice per month (2)
    - Three times per month (3)
    - Four time per month (4)
    - More than four times per month, please specify (5) ------------
    - Never (6)

Display this question: If Does your child ever stress dose for psychological/emotional situations such as feeling depressed... = Yes, please specify

- **Did your child's provider advise you to do this? Please provide any other details you would like to share.**
    - Yes, please specify (1) ____
    - No, please specify (2) ____

- **Do you have a written instruction guideline sheet for stress-dosing during adrenal crisis via an injection that was given to you by your child's physician?**
    - Yes (1)
    - No (2)

Start of Block: Quality of Life for Parent Response

This survey asks about health experiences over the past 4 weeks and throughout life in general. Please answer each question based on your first instinct—your immediate response is often the most accurate. If you are a parent or guardian completing this survey on behalf of your child, please answer to the best of your knowledge. If you are unsure or feel uncomfortable with any question, you may skip it. Thank you for your participation!

- **Please choose one answer that describes your child's health related experiences in the past 4 weeks.**

	Never (1)	A little of the time (2)	Sometimes (3)	Often (4)	Always (5)
My child feels good about their health. (1)	o	o	o	o	o
My child worries about their health. (2)	o	o	o	o	o
My child misses more school than their peers due to illness. (3)	o	o	o	o	o
My child gets ill more easily than others. (4)	o	o	o	o	o
My child takes a long time to recover from illnesses. (5)	o	o	o	o	o

- **Please choose one answer that describes your child's energy related experiences in the past 4 weeks.**

	Never (1)	A little of the time (2)	Sometimes (3)	Often (4)	Always (5)
My child feels full of energy. (1)	o	o	o	o	o
Normal daily activities makes my child tired. (2)	o	o	o	o	o
My child feels rested when they wake up in the morning. (3)	o	o	o	o	o
My child feels unwell first thing in the morning. (4)	o	o	o	o	o
My child gets nauseous. (5)	o	o	o	o	o
My child feels lightheaded. (6)	o	o	o	o	o
My child can't keep going throughout the day without feeling tired. (7)	o	o	o	o	o
My child craves salt more than the average person. (8)	o	o	o	o	o

My child has joint or bone pain. (9)	○	○	○	○	○

- **Please choose one answer that describes your child's nutrition related experiences in the past 4 weeks.**

	Never (5)	A little of the time (4)	Sometimes (3)	Often (2)	Always (1)
Stretch marks have been a problem for my child. (1)	○	○	○	○	○
My child is afraid to fall and suffer a bone fracture. (2)	○	○	○	○	○
My child is careful not to crash into things because they bruise easily. (3)	○	○	○	○	○
My child worries about gaining weight. (4)	○	○	○	○	○
My child does not feel they have had enough food even after an adequate meal portion. (5)	○	○	○	○	○
My child eats the same amount or less than other family members, but they are still gaining weight. (6)	○	○	○	○	○

- **Please choose one answer that describes your child's activity related experiences in the past 4 weeks.**

	Never (1)	A little of the time (2)	Sometimes (3)	Often (4)	Always (5)
Is your child able to exercise or play sports in the heat? (1)	○	○	○	○	○
Is your child able to maintain hydration while exercising? (2)	○	○	○	○	○
Is your child able to do moderate activities (brisk walk, vacuuming, leisure biking)? (3)	○	○	○	○	○
Is your child able to do vigorous activities such as running, lifting heavy objects, or participating in strenuous sports? (4)	○	○	○	○	○

Is your child able to recover quickly after exercising? (5)	○	○	○	○	○
Does your child express feeling physically fit. (6)	○	○	○	○	○

- **Please choose one answer that describes your child's health related experiences in the past 4 weeks.**

	Never (5)	A little of the time (4)	Sometimes (3)	Often (2)	Always (1)
My child has expressed or presented with trouble falling asleep. (1)	○	○	○	○	○
My child has expressed or presented as irritable. (2)	○	○	○	○	○
My child has expressed or presented with difficulty relaxing. (3)	○	○	○	○	○
My child has expressed feeling anxious. (4)	○	○	○	○	○
My child has expressed feeling low or depressed. (5)	○	○	○	○	○
My child has expressed or presented with difficulty concentrating. (6)	○	○	○	○	○
My child has expressed or presented with difficulty remembering. (7)	○	○	○	○	○
My child has expressed or presented with slow mental and motor activity. (8)	○	○	○	○	○
My child has expressed or presented with low self-confidence. (9)	○	○	○	○	○

Start of Block: Additional questions for parents/care givers only

- **Has your child expressed challenging experiences related to CAH? --------------**
- **What do you want your child’s doctor to know about what it’s like for your child to live with CAH? ------------------------**

Start of Block: Gender_Sex_patient responce

- **What is your sex?**
  - ○ Male XY (1)
  - ○ Female XX (2)

- Prefer not to say (4)

Start of Block: Provider_and_Resources for patient

- **Do you see an endocrinologist to manage and treat Congenital Adrenal Hyperplasia (CAH)?**
  - Yes (1)
  - No (2)

Display this question: If Do you see an endocrinologist to manage and treat Congenital Adrenal Hyperplasia (CAH)? = Yes

- **If yes, do you consider your provider to be an expert in CAH management?**
  - Yes (1)
  - No (2)

Display this question: If Do you see an endocrinologist to manage and treat Congenital Adrenal Hyperplasia (CAH)? = Yes

- **Approximately how many miles do you travel to see your endocrinologist? -----**

Display this question: If Do you see an endocrinologist to manage and treat Congenital Adrenal Hyperplasia (CAH)? = No

- **If no, what type of healthcare provider treats your CAH?**
  - General pediatrician (1)
  - Family practice physician (2)
  - Nurse practitioner (3)
  - Other (4) ____

- **Is the health care provider who manages and treats your CAH affiliated with a CARES Comprehensive Care Center? If so please list them.**
  - Yes (1)
  - No (2)

Display this question: If Is the health care provider who manages and treats your CAH affiliated with a CARES Comprehensive... = Yes

- **If you selected yes, please chose the facility or facilities they are affiliated with.**
  - New York-Presbyterian/Weill Cornell Medical Center (1)
  - Children's Hospital Los Angeles / Keck School of Medicine (2)
  - Riley Hospital for Children/ Indiana University Health – Indianapolis IN (3)
  - Rutgers-Robert Wood Johnson Medical School (RWJMS), Child Health Center of New Jersey (CHINJ) (4)
  - Children's Health Medical Center/UT Southwestern Medical Center – Dallas, Texas (5)

- Children's Hospital of Philadelphia/Penn Med – Philadelphia, PA (6)
- Seattle Children's Hospital and University of Washington Medical Center – Seattle, Washington (7)
- Cook Children's Health Care System – Fort Worth, Texas (8)
- Other (9) ____

- **How far from your home is the hospital where you would go if you were in an adrenal crisis?**
  - Less than 5 miles (Less than 8 km) (1)
  - 5–10 miles (8–16 km) (2)
  - 10–20 miles (16–32 km) (4)
  - 20–50 miles (32–80 km) (5)
  - More than 50 miles (More than 80 km) Please specify (6) ____

Start of Block: Geography_and_finance_Patient_responce

- **Where do you live (city/state/country or province/territory/city/country)?**
  - United States US, please specify (1) -------------
  - Outside of United states US, please specify (2) --------------

Display this question: If Where do you live (city/state/country or province/territory/city/country)? = United States US, please specify

- **Approximate gross yearly family income (before taxes):**
  - Less than $20,000 (1)
  - $20,000 to $29,999 (2)
  - $30,000 to $39,999 (3)
  - $40,000 to $49,999 (4)
  - $50,000 to $59,999 (5)
  - $60,000 to $69,999 (6)
  - $70,000 to $79,999 (7)
  - $80,000 to $89,999 (8)
  - $90,000 to $99,999 (9)
  - $100,000 or more (10)
  - I'm not sure (11)

- **How do you pay for your medical care, choose all that apply?**

- Private insurance through your job (1)
- National Health Care (ex. UK and much of Europe) (2)
- Medicare/Medicaid (US) (3)
- Marketplace/Affordable Care Act (US) (4)
- Other (5) ____

Start of Block: Medical Information_ patient

- **To the best of your knowledge, how many times per year do you have an adrenal crisis?** ____
- **Have you had a Bone density or DEXA scan?**
  - Yes (1)
  - No (2)

Display this question: If Have you had a Bone density or DEXA scan? = Yes

- **Have you been told you have problems with your bones (ex. osteoporosis/osteopenia/bone fractures)?**
  - Yes, please specify (2) ____
  - No (4)
- **Do you have any other medical conditions, including but not limited to hypertension, high blood pressure, or diabetes? If yes, please list them below.**
  - Yes, please specify (4) ____
  - No (5)
- **Please list all the medications you are currently taking related to your CAH (eg fludrocortisone/Florinef, hydrocortisone/Cortef/Alkindi, prednisone, crinecerfont/Crenessity). ----------**

Start of Block: Adult_CAH_Questions

Display this question: If What is your sex? = Female XX

- **Have you struggled with fertility? If yes, please describe.**
  - Yes, please specify. (1) ____
  - No (2)

Display this question: If What is your sex? = Female XX

- **Have you given birth? If yes, did you use reproductive assistance such as IVF, IUI, or medication?**
  - Yes, please specify. (1) ____

- No (2)

Display this question: If What is your sex? = Female XX

- **Have you had genital surgery? If yes, at what age?**
  - Yes, please specify. (1) ____
  - No (2)

Display this question: If What is your sex? = Male XY

- **Have you obtained a testicular ultrasound (U/S), if yes, how often (XY males only)?**
  - Yes, please specify (1) ____
  - No (2)

Display this question: If What is your sex? = Male XY

- **Do you have Testicular Adrenal Rest Tumors (TART).**
  - Yes (1)
  - No (2)

Start of Block: Solu-Cortef_ patient

- **Do you have a prescription for injectable hydrocortisone (also known as Solu-Cortef)?**
  - Yes (1)
  - No (2)

Skip To: End of Block If Do you have a prescription for injectable hydrocortisone (also known as Solu-Cortef)? = No

- **Which statement best fits how you obtained your prescription for injectable hydrocortisone?**
  - My doctor prescribed it at diagnosis without me saying anything (1)
  - Sometime after diagnosis I had to ask my physician for the prescription of injectable hydrocortisone (2)
  - My physician prescribed injectable hydrocortisone for me eventually without my asking for it (3)
  - You had to change physicians in order to receive the prescription for injectable hydrocortisone (4)
  - other (5) ____

- **Has your physician OR one of their staff (such as a nurse) ever demonstrated to you how to correctly administer a shot of injectable hydrocortisone to yourself?**
  - Yes (1)
  - No (2)
  - I cant remember (3)

- **Have you filled your Solu-Cortef prescription? If not, what was the reason? (ex. I didn't want to use it, I didn't have transportation, I didn't know how to use it)?**
    - o Yes (1)
    - o No, please specify (2) ____
- **Do you consistently have a vial of injectable hydrocortisone readily available for an emergency?(for example, work, in the home, etc.)**
    - o Definitely not (1)
    - o Probably not (2)
    - o Might or might not (3)
    - o Probably yes (4)
    - o Definitely yes (5)
- **Do you ever stress dose for psychological/emotional situations such as feeling depressed or experiencing a life changing event (death, illness in a family member)? If yes please list them?**
    - o Yes, please specify (2) ____
    - o No (3)

Display this question: If Do you ever stress dose for psychological/emotional situations such as feeling depressed or exper... = Yes, please specify

- **If so, how many times per month would you say you do this? If more than four times, please describe.**
    - o Once a month (1)
    - o Twice per month (2)
    - o Three times per month (3)
    - o Four time per month (4)
    - o More than four times per month, please specify (5) -------------
    - o Never (6)

Display this question: If Do you ever stress dose for psychological/emotional situations such as feeling depressed or exper... = Yes, please specify

- **Did your provider advise you to do this? Please provide any other details you would like to share.**
    - o Yes, please specify (1) ____
    - o No, please specify (2) ____
- **Do you have a written instruction guideline sheet for stress-dosing during adrenal crisis via an injection that was given to you by your physician?**

- Yes (1)
- No (2)

Start of Block: Quality of Life_Patient Responce

This survey asks about health experiences over the past 4 weeks and throughout life in general. Please answer each question based on your first instinct—your immediate response is often the most accurate. If you are unsure or feel uncomfortable with any question, you may skip it. Thank you for your participation!

- **Please choose one answer that describes your health related experiences in the past 4 weeks.**

	Never (1)	Sometimes (2)	About half the time (3)	Most of the time (4)	Always (5)
I feel good about my health. (1)	o	o	o	o	o
I worry about my health. (2)	o	o	o	o	o
I miss more school or work than my peers due to illness. (3)	o	o	o	o	o
I get ill more easily than others. (4)	o	o	o	o	o
I take a long time to recover from illnesses. (5)	o	o	o	o	o

- **Please choose one answer that describes your energy related experiences in the past 4 weeks.**

	Never (1)	Sometimes (2)	About half the time (3)	Most of the time (4)	Always (5)
I feel full of energy. (1)	o	o	o	o	o
Normal daily activities make me tired. (2)	o	o	o	o	o
I feel rested when I wake up in the morning. (3)	o	o	o	o	o
I feel unwell first thing in the morning. (4)	o	o	o	o	o
I get nauseous. (5)	o	o	o	o	o
I feel lightheaded. (6)	o	o	o	o	o
I can keep going throughout the day without feeling tired. (7)	o	o	o	o	o
I crave salt more than the average person. (8)	o	o	o	o	o
I have joint or bone pain. (9)	o	o	o	o	o

- **Please choose one answer that describes your nutrition related experiences in the past 4 weeks.**

	Never (5)	Sometimes (6)	About half the time (7)	Most of the time (8)	Always (9)
Stretch marks have been a problem for me. (1)	o	o	o	o	o
I am afraid to fall and suffer a bone fracture. (2)	o	o	o	o	o
I watch out to not crash into things because I get bruises easily. (3)	o	o	o	o	o
I worry about gaining weight. (4)	o	o	o	o	o
I do not feel I have had enough food even after an adequate meal portion. (5)	o	o	o	o	o
I eat the same amount or less than other family members, but I am still gaining weight. (6)	o	o	o	o	o

- **Please choose one answer that describes your activity related experiences in the past 4 weeks.**

	Never (1)	Sometimes (2)	About half the time (3)	Most of the time (4)	Always (5)
Are you able to exercise or play sports in the heat? (1)	o	o	o	o	o
Are you able to maintain hydration while exercising? (2)	o	o	o	o	o
Are you able to do moderate activities (brisk walk, vacuuming, leisure biking)? (3)	o	o	o	o	o
Are you able to do vigorous activities such as running, lifting heavy objects, or participating in strenuous sports? (4)	o	o	o	o	o
Are you able to recover quickly after exercising? (5)	o	o	o	o	o
I feel physically fit. (6)	o	o	o	o	o

- **Please choose one answer that describes your stress related experiences in the past 4 weeks.**

	Never (1)	Sometimes (2)	About half the time (3)	Most of the time (4)	Always (5)
I have trouble falling asleep. (1)	o	o	o	o	o
I am irritable. (2)	o	o	o	o	o
I am relaxed. (3)	o	o	o	o	o
I am anxious. (4)	o	o	o	o	o
I feel low or depressed. (5)	o	o	o	o	o
I can concentrate well. (6)	o	o	o	o	o
I forget things. (7)	o	o	o	o	o

I am slowed in my mental and motor activity. (8)	○	○	○	○	○
I am self-confident. (9)	○	○	○	○	○

- **Please choose one answer that describes your social experience in the past 4 weeks.**

	Never (5)	Sometimes (4)	About half the time (3)	Most of the time (2)	Always (1)
I have trouble making friends. (1)	○	○	○	○	○
Friends and/or family members treat me differently than other people my age. (2)	○	○	○	○	○
I feel uncomfortable going to school or work. (3)	○	○	○	○	○
I feel uncomfortable about sharing certain things about having CAH with people around me (for example taking medications)? (4)	○	○	○	○	○
My health or emotional problems interfered with my social activities (like visiting friends, relatives, etc.). (5)	○	○	○	○	○

- **Please choose one answer that describes your intimate experiences in the past 4 weeks.**

	Never (5)	Sometimes (4)	About half the time (3)	Most of the time (2)	Always (1)
I am troubled by the appearance of my genitalia. (1)	○	○	○	○	○
I have anxiety or distress about sexual activity. (2)	○	○	○	○	○
I have less sexual desire or interest. (3)	○	○	○	○	○
I'm dissatisfied with my overall sexual life. (4)	○	○	○	○	○

Start of Block: Additional Questions for_person_living_w/_CAH

- **What is the biggest challenge related to having CAH? -------------------**
- **What do you wish your healthcare provider knew about your life with CAH? _____________**
